# An Effective Assessment of Simvastatin-Induced Toxicity with NMR-Based Metabonomics Approach

**DOI:** 10.1371/journal.pone.0016641

**Published:** 2011-02-22

**Authors:** Hye-ji Yang, Myung-Joo Choi, He Wen, Hyuk Nam Kwon, Kyung Hee Jung, Sang-Won Hong, Joon Mee Kim, Soon-Sun Hong, Sunghyouk Park

**Affiliations:** 1 Department of Biochemistry, Inha University Hospital and Center for Advanced Medical Education by BK21 project, College of Medicine, Inha University, Incheon, Korea; 2 Department of Biomedical Sciences, Inha University Hospital and Center for Advanced Medical Education by BK21 project, College of Medicine, Inha University, Incheon, Korea; 3 Department of Pathology, Inha University Hospital and Center for Advanced Medical Education by BK21 project, College of Medicine, Inha University, Incheon, Korea; University of Illinois at Chicago, United States of America

## Abstract

**Background:**

Simvastatin, which is used to control elevated cholesterol levels, is one of the most widely prescribed drugs. However, a daily excessive dose can induce drug-toxicity, especially in muscle and liver. Current markers for toxicity reflect mostly the late stages of tissue damage; thus, more efficient methods of toxicity evaluation are desired.

**Methodology/Principal Findings:**

As a new way to evaluate toxicity, we performed NMR-based metabonomics analysis of urine samples. Compared to conventional markers, such as AST, ALT, and CK, the urine metabolic profile provided clearer distinction between the pre- and post-treatment groups treated with toxic levels of simvastatin. Through multivariate statistical analysis, we identified marker metabolites associated with the toxicity. Importantly, we observed that the treatment group could be further categorized into two subgroups based on the NMR profiles: weak toxicity (WT) and high toxicity (HT). The distinction between these two groups was confirmed by the enzyme values and histopathological exams. Time-dependent studies showed that the toxicity at 10 days could be reliably predicted from the metabolic profiles at 6 days.

**Conclusions/Significance:**

This metabonomics approach may provide a non-invasive and effective way to evaluate the simvastatin-induced toxicity in a manner that can complement current measures. The approach is expected to find broader application in other drug-induced toxicity assessments.

## Introduction

Simvastatin is among the most prescribed drugs in western countries, and reduces morbidity and mortality from coronary heart diseases [1]. It inhibits the enzyme 3-hydroxy-3-methylglutaryl co-enzyme A (HMG-CoA) reductase, the rate-limiting step in cholesterol biosynthesis [2], [3], [4]. The inhibition of HMG-CoA reductase induces depletion of intracellular sterols, up-regulating low-density lipoprotein (LDL) receptors, principally in the liver, with subsequent increased uptake of cholesterol-containing lipoproteins. In addition to their lowering effects on cholesterol levels, a number of other clinical benefits of statins have been recognized [5].

Although statins are generally well tolerated, they do have side-effects. The most common adverse reaction is myopathy [6]. The clinical manifestations of statin-associated myopathy include pain and muscle weakness, with a prevalence of 10%–15% [7]. While the mechanisms of simvastatin-induced myopathy have not been fully elucidated, it is likely that simvastatin induces myopathy by disrupting isoprenoid intermediates in the cholesterol synthesis pathway. Its effects on muscle range in severity from myalgia and limb weakness to myopathy, often accompanied by elevated serum creatinine kinase (CK), or more pronounced skeletal muscle breakdown, in which the release of myoglobin can cause renal damage. It has been reported that the development of myopathy follows a characteristic pattern of elevated serum CK and skeletal muscle necrosis [8], [9], [10].

Simvastatin has also been reported to cause adverse effects in liver due to cellular damage. The incidence of liver function abnormality increases approximately 4- to 5-fold with increasing dose of simvastatin [11]. In addition, Clarke et al. reported that simvastatin might cause hepatitis, cholestatic jaundice, cirrhosis, hepatic failure, and hepatic necrosis in some patients [12]. In this report, atorvastatin and pravastatin also caused similar adverse effects with transient increases in serum transaminases. However, there are relatively few studies on liver toxicity and the associated mechanism by statin treatment.

Metabonomics is a global metabolite profiling approach for biological samples, particularly, biofluids. Since it involves a large quantity of data, it is often combined with multivariate statistical analysis in order to efficiently assess principal factors contributing to the phenotypic changes. It can be readily applied to monitor the changes in metabolite concentration and profiles in response to non-physiologic challenges such as drugs or toxins [13], [14]. Such studies can also provide information about the sites and basic mechanism of toxicity, as well as potential metabolic biomarkers [15] which can be used for safety evaluation processes [16]. Recently, metabonomics techniques have shown its utility in predicting drug-induced toxicity based on pre-dose metabolic profiles [17], [18]. For metabonomics studies, it is desirable to obtain broad coverage of the metabonome to facilitate the discovery of potential biomarkers. Therefore, ^1^H Nuclear Magnetic Resonance (NMR) spectroscopy of biofluids has been the method of choice due to its superb reproducibility and quantitativeness [19], [20], [21], [22]. In terms of biofluid samples, urine is one of the major targets, as it is a complex biological sample containing a large number of endogenous metabolites reflecting the metabolic state of an organism [23]. In addition, urine collection is both simple and non-invasive, making it a preferred sample for monitoring metabolic biomarkers.

In this study, we evaluated the applicability of the NMR-based metabonomics approach in assessing simvastatin-induced toxicity. We also sought to identify non-invasive urinary biomarkers. Our results showed that ^1^H-NMR-based metabonomics approach is significant and sensitive enough to complement conventional biochemical enzyme markers (AST, ALT, and CK) in evaluating the side effects of simvastatin.

## Results

### Serum biochemical parameters

**Figure 1:**
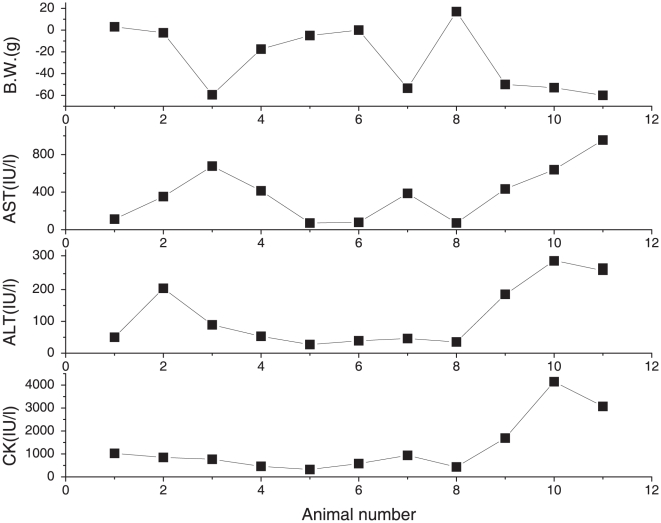
Changes of body weight and biochemical parameters of each rat after simvastatin treatment. Blood samples were collected after 10 days of simvastatin treatment (80 mg/kg). Serum alanine transaminase (ALT), aspartate transaminase (AST), and plasma creatinine kinase (CK) activities were measured using commercial kits employing spectrophotometric assays. All biomarkers were analyzed at Inha University Hospital (Incheon, Korea).

To investigate the toxic effects of simvastatin, we first performed well-established biochemical enzyme tests for AST, ALT, and CK. We selected these enzymes because simvastatin-induced toxicity has been reported for liver and muscle. The values from the untreated group were as follows: AST, 107.50±7.78; ALT, 29.67±1.91; CK, 789.67±52.10, which are within normal ranges routinely observed in our lab (See Table S1 and S2). Compared with these values, the AST activity was increased by 2.7-fold (284.17±87.58, p<0.02) and the ALT activity by 4.0-fold (115.73±175.57, p<0.03) in the simvastatin-treated group indicating drug-induced liver toxicity. In comparison, the CK level, which is often associated with muscle toxicity in humans, was not significantly different (1298.82±396.52, p>0.16). However, for all three enzymes, there were large variations among the individual animals. Some animals showed pronounced toxicity, while others exhibited relatively low toxicity, and the trends did not match among the enzyme classes (Fig. 1). For example, simvastatin treatment seemed to be highly toxic to some subjects (A-9, A-10, A-11) in all four factors, while it seemed to be weakly toxic to others (A-1, A-5, A-6, A-8) (See Fig. 1). In addition, some subjects showed quite different results depending on the criteria. For example, A-3 showed highly toxic response in terms of the change of body weight and AST, but it showed only weak toxicity in terms of ALT and CK. In addition, the values obtained from simvastatin-treated animals were much more variable within each of the enzyme classes. Therefore, it was not immediately clear which enzyme classes or values should be used to decide the toxicity manifestation.

### NMR Spectroscopy of urine samples

**Figure 2:**
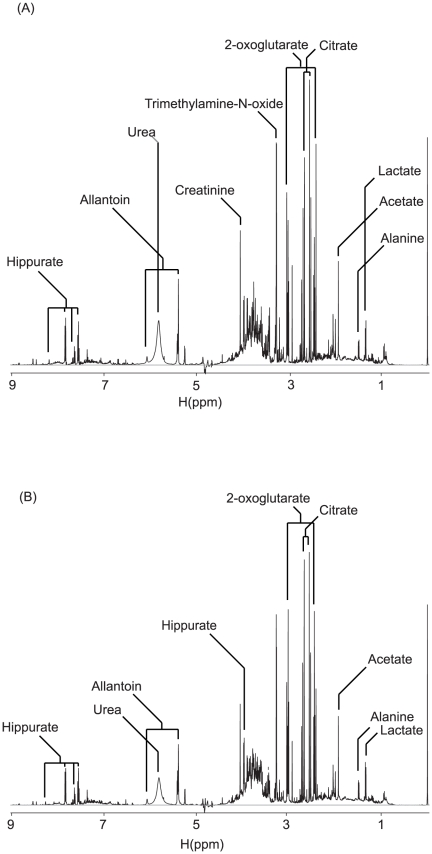
Representative ^1^H NMR spectra of urine from animals before and after simvastatin treatment. The spectrum before the simvastatin treatment is at the top, and that after the treatment is at the bottom. Metabolite peaks were assigned using Chenomx (Spectral database; Edmonton, Alberta, Canada). The spectra were taken for urine samples containing 150 mM phosphate (pH 7.4) and 0.025% TSP as a chemical shift reference.

With the variable results from the biochemical data, we explored toxicity evaluation by metabonomics using urine samples. Nuclear magnetic resonance (NMR)-based metabonomic analysis of urine samples offers several advantages in that urine samples can be obtained non-invasively and that they reflect more systemic effects than individual biochemical enzymes [14], [24], [25]. Moreover, NMR spectroscopy can give structural information about the potential biomarkers. Representative ^1^H NMR spectra of urine from animals before and after simvastatin treatment are shown in Fig. 2. We identified a number of constituents in the urine as a preliminary step in finding marker metabolites. Although there were some apparent differences between these representative spectra based on simple visual inspection, they were not consistent across all samples. Therefore, we applied a multivariate statistical method to analyze the spectra in a more holistic way and to identify signals that can efficiently differentiate the groups.

### OPLS-DA Multivariate analysis

**Figure 3:**
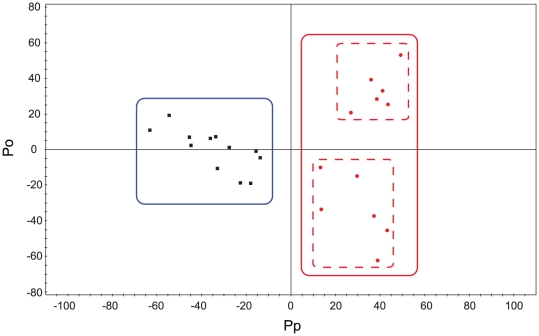
Differentiation of pre- and post-treatment groups using multivariate analysis. Orthogonal projections to latent structure-discriminant analysis (OPLS-DA) score plot of the pre- and post-groups. For the post-treatment group, urine samples collected after 10 days of treatment were used. Black squares: pre-treatment samples; Red circles: post-treatment samples. The model was established using one predictive and one orthogonal component.

Since we observed intra-group variation, we analyzed the data with the OPLS-DA multivariate approach, which can separate groups in the presence of large structured noise [26], [27]. The differentiation model for distinguishing the animals before (pre-group) and after (post-group) simvastatin treatment was built using one predictive and four orthogonal components (Fig. 3). The model had an overall goodness of fit, R^2^(Y), of 96% and an overall cross-validation coefficient, Q^2^(Y), of 68%. Out of the overall R^2^(X) value of 0.83, 63% was structured, but uncorrelated to the response, and 20% was predictive, that is, responsible for the class separation. The resulting score plot shows that the pre- and post-groups can be clearly differentiated by the first predictive component derived from the NMR spectral variables. This differentiation was thought due to the liver toxicity, as the average values of AST and ALT were statistically higher in the simvastatin treated group (see Table S1).

**Figure 4:**
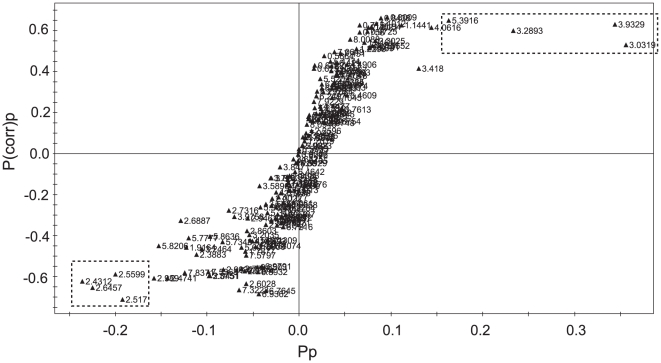
Signals contributing to the differentiation of pre- and post-treatment groups. S-plot analysis representing the highest contributing signals for the pre- and post-groups. The p_p_ represents modeled covariation, and p(corr)_p_ represents modeled correlation. Potential marker signals that are significantly biased across the two groups are enclosed in dotted boxes.

**Figure 5:**
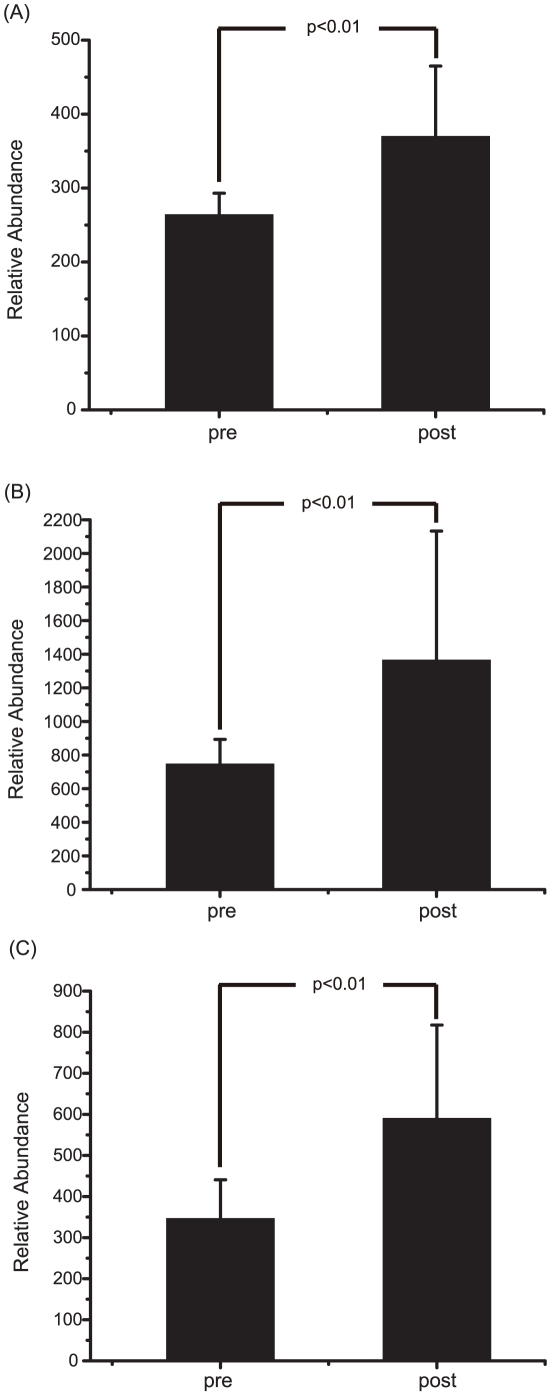
Levels of the marker signals in the pre- and post-treatment groups. Student's *t*-test of the relative distribution of the marker signals for the pre- and post-treatment group. The resulting p-values are indicated, and all the signals showed statistical significance with p<0.01. (A) Top: 5.3916 ppm; (B) Middle: 3.0319 ppm; (C) Bottom: 3.2893 ppm.

One advantage of the metabonomics approach is that it can give marker metabolites that contribute to the differentiation. Therefore, we explored the orthogonal partial least-squares discrimination analysis (OPLS-DA) model to identify the metabolites that are characteristic of the group with toxic responses. We constructed an S-plot that can show the modeled covariation, p_p_, with the modeled correlation, p(corr)_p_, in one graph. The resulting S-plot shows that most of the markers for the post-group have signals in the 3.0–5.4 ppm region (Fig. 4). In contrast, the markers for the pre-group have signals in the 2.4–2.6 ppm region. Based on the urine constituents identified above, the marker signals for the post-groups belong to 2-oxoglutarate (3.03 ppm), trimethylamine-N-oxide (3.29 ppm) and allantoin (5.39 ppm). To demonstrate the actual biased distribution of the toxicity markers found by the multivariate analysis, we compared the relative amounts of the markers in both of the groups. Fig. 5 shows that the levels of the toxicity markers were significantly higher in the post-group. All of the markers showed strong statistical significance with p-values of less than 0.01 obtained from Student's *t*-test.

### Different toxic responses within the post-group

**Figure 6:**
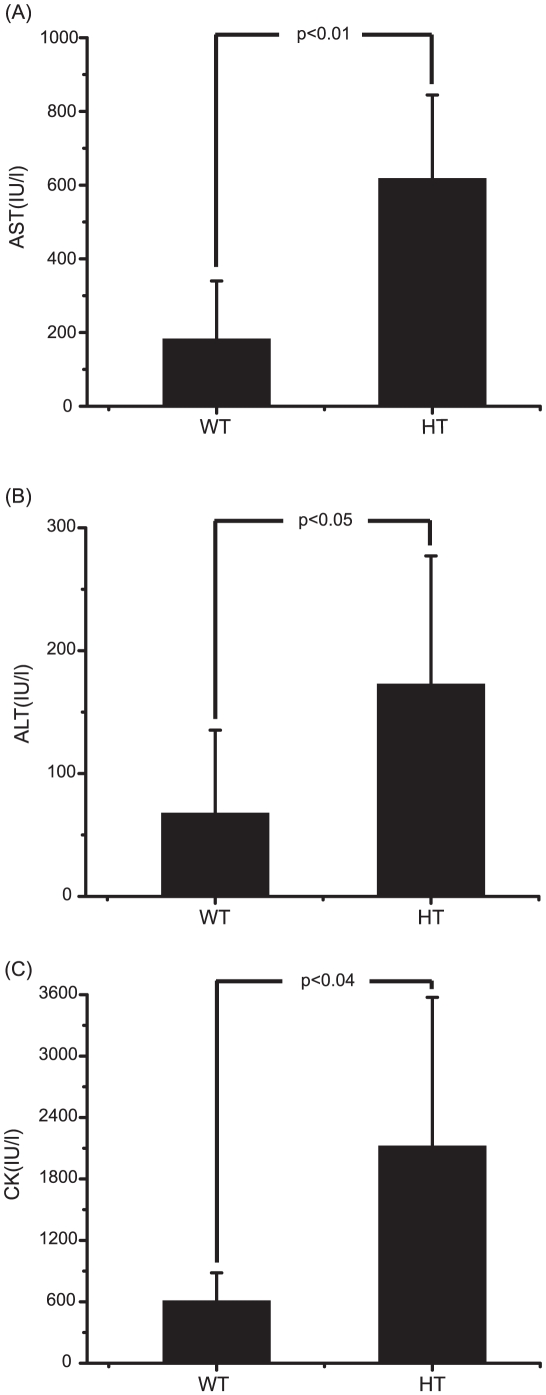
Difference between WT and HT subgroup: biochemical data. The WT and HT subgroups among the simvastatin treated animals were categorized according to the OPLS-DA model as described in the text. Average values of AST, ALT and CK levels of each group after 10 days of treatment are plotted along with the standard deviation. Student's *t*-test was also performed and the resulting p values are also indicated. (A) AST; (B) ALT; (C) CK.

In examining the NMR multivariate data in more detail, we found that the post-group can be divided into two subgroups along the Y axis of the plot (See the dotted red lines in Fig. 3), whereas the pre-group is clustered tightly without such intra-group separation. This indicates that there could be differences in the individual toxicity levels in the post-group. To examine this possibility, we re-analyzed the biochemical enzyme data for these two subgroups. Fig. 6 shows that there are significant differences in the enzyme values between the two subgroups, consistent with the NMR-based analysis. In addition, the subgroup with higher CK values showed significantly different values from the control group, despite the fact that the post-group as a whole did not show such difference (Table S1). Based on the results, we designated the subgroups as WT and HT groups.

### Histopathological confirmation of the toxicity subgroups

**Figure 7:**
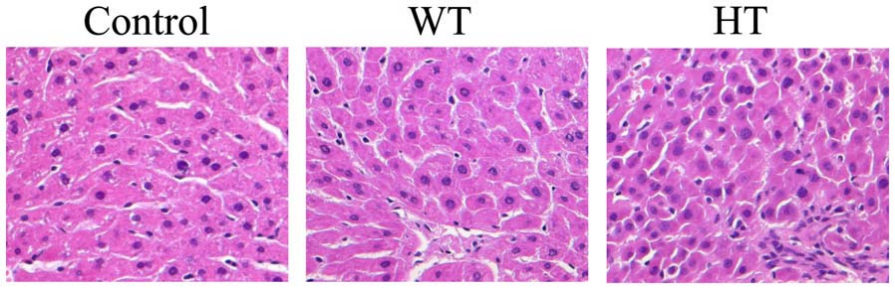
Histopathological analysis of liver sections. The liver tissues were collected after 10 days of simvastatin treatment. The tissues were fixed in 10% formaldehyde, and then stained using Hematoxylin and Eosin (H&E). Original magnification ×400. Control, control group; WT, weak toxicity group; HT, high toxicity group.

To examine the difference in the toxicity at the tissue level, we obtained the histopathological data on liver tissues. H&E staining of the liver showed that the control group had mostly intact nuclei and normal cell shapes. In addition, the hepatic lobular structure and portal tract were well preserved without inflammation or necrosis (Fig. 7). The lobules in the HT group, however, showed increased Kupffer cell density and inflammatory cell infiltration. Moreover, the hepatocytes showed occasional acidophilic degeneration, necrosis or swelling, and regeneration activity, which suggest significant cellular damage. In comparison, the WT group showed similar characteristics in lobular structures and inflammation status to the control group. These data validate the categorization of the post-group into two subgroups and prove the effectiveness of the NMR metabonomics approach.

### Time dependent toxicity according to urine NMR analysis

**Figure 8:**
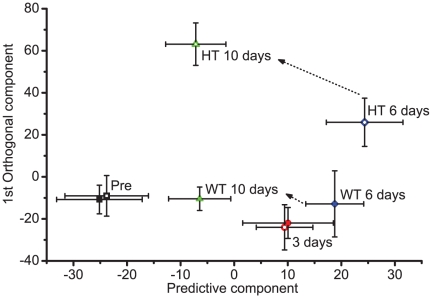
Time course of the urine metabolite profile. Urines collected at specified time points during the simvastatin treatment (80 mg/kg) were analyzed by NMR and OPLS-DA. The coordinates of each time point represent the average score values from the multivariate analysis. The whiskers represent one standard deviation. Open symbols represent the HT group and the filled symbols WT group. Each time point is represented by different colored symbols: Pre, black squares; 3 Day, red circles; 6 Day, blue diamonds; 10 Day, green triangles. The transitions for the 6 day to 10 day are specified by arrows. Others are not indicated to simplify the figure, but can be easily traced by following the open or filled symbols, respectively.

To investigate how effectively these subgroup differences can be detected, we obtained urine at several time points and analyzed the corresponding NMR spectral data. The results in Fig. 8 show that the difference is not clear before or 3 days after the drug treatment (black or red symbols, respectively). In comparison, the WT and HT group diverged from 6 days after the treatment, following different paths thereafter and ending up in very distinct parts of the two-dimensional reduced-dimension OPLS space. These analyses show that NMR data alone can differentiate between WT and HT groups as early as 6 days after the treatment. Equally interestingly, after 10 days, the Euclidean distance between the WT and pre-group was much smaller than that between the HT and pre-group. In addition, the final coordinate of the WT group after 10 days was even closer to the pre-group than those after 3 and 6 days. These findings suggest that the urine profile of the WT group after 10 days is more similar to that from pre-group than that from the 3 or 6 days, consistent with the biochemical toxicity and histopathological results.

## Discussion

Simvastatin is relatively safe in normal dosage. In previous case studies, however, it has been reported to be hepatotoxic and myotoxic in cumulative high doses. In order to investigate the toxic effects of simvastatin, we first performed well-established biochemical enzyme tests for AST, ALT, and CK. In terms of the evaluation of toxicity, the values from just one or two biological surrogate serum enzymes were not decisive. In our results, the changes in body weight, AST, ALT, and CK were not correlated with each other in all subjects. Therefore, it was not easy to obtain consistent indications regarding the toxicity of simvastatin in these subjects. In comparison, the metabonomics studies provided a clearer distinction of the toxicity, new toxicity markers, and further differentiation of the WT and HT groups.

From the analysis of NMR patterns before and after treatment with simvastatin, three new biological markers were discovered: allantoin, 2-oxoglutarate, and trimethylamine-N-oxide. These markers were all increased by simvastatin treatment. Allantoin is formed by nonenzymatic oxidation of urate, and is reported to be a possible indicator of free radical damage [28]. Therefore, higher levels of allantoin in the HT group suggest that simvastatin may induce oxidative stress. 2-oxoglutarate is generated from isocitrate through oxidative decarboxylation in the tricarboxylic acid cycle. Another round of oxidative decarboxylation transforms it into succinate and also generates GTP. Both of these decarboxylation steps generate NADH, a key molecule in oxidation-reduction reactions. Therefore, 2-oxoglutarate is intimately involved in the cellular detoxification of oxidative damage. The changes in its level in the HT group suggest that the observed simvastatin-induced toxicity is related to oxidative stress, consistent with our interpretation of the changes in allantoin levels. Trimethylamine-N-oxide (TMAO) is an oxidation product of trimethylamine and is also involved in the oxidation-reduction reactions for nucleotide metabolism. High levels of TMAO have been implicated in inflammatory responses present in Rheumatoid arthritis [29]. Overall, these markers suggest that the simvastatin-induced toxicity seems to mainly stem from oxidative stress-related inflammatory responses. In this sense, it is interesting to note that a similar drug, atorvastatin, increased the urine levels of proline and 3-ureidopropionic acid that are associated with oxidative toxicity in rat system [30] [31], [32].

For the specific oxidative pathway, simvastatin might decrease the prodution of Coenzyme Q10 (CoQ10). This is feasible because simvastatin inhibits HMG CoA reductase that generates mevalonate which, in turn, is a precursor for CoQ10. CoQ10, as an antioxidant, has membrane stabilizing effects and is important for cellular mitochondrial respiration, essential for energy production in organs[33], [34]. A previous study showed that simvastatin induced DNA oxidative stress in liver cells and, CoQ10 treatment protected liver cells from simvastatin's toxic effect [35]. In addition, CoQ10 treatment improved oxidative stress response by increasing NADPH-CoQ reducates in liver injury induced by simvastatin [36]. These results are consistent with the possible involvement of simvastatin in the antioxidative pathway of CoQ10.

Our results provide several practical applications of the NMR-based metabonomics approach in evaluating simvastatin toxicity. First, as the presence of the WT and HT subgroups and their difference from the pre-group were not immediately apparent from only the biochemical data, the metabonomics approach seems to allow more detailed assessment of the toxicity than conventional markers. It should be noted that the urine profile measured by NMR not only distinguished between the subgroups, but also gave a measure of the degree of toxicity in the forms of the direction and the Euclidean distance of a particular group from the control group. The validity of the results was confirmed by its consistency with the biochemical and histopathological data. Second, the metabonomics data may allow earlier detection of the toxicity, enabling the prediction of later toxicity. Our data showed that the toxicity at 10 days of treatment was reliably correlated with that at 6 days. As the HT group showed more pronounced toxicity than the WT group, the urine metabolite profile might be used to decide whether drug treatment should be continued or not, before it causes significant and/or irreversible damages. Third, the NMR urine profile can provide toxicity data in a completely non-invasive manner. Although the histopathological method is the gold standard in toxicity assessment, it requires an invasive biopsy and can sometimes cause unintended complications, for example, infections during the sampling. Although the AST, ALT, and CK values can be measured in serum samples, which are considered minimally invasive, the blood can only be collected in a hospital setting by appointment with specialized personnel. In contrast, urine can be collected without these limitations. Therefore, the metabonomics approach has added convenience in addition to its merits in toxicity assessment.

Taken together, NMR-based metabolite profiling combined with multivariate analysis may provide new criteria for evaluating the simvastatin-induced toxicity that can complement currently available biochemical or histopathological measures. With the convenience of sample collection, the possibility of predicting the future responses, and the technical robustness, this approach is expected to find broader applications in other drug-induced toxicity assessments.

## Materials and Methods

### Materials

Simvastatin USP was obtained from Biocon (Bangalore, India). Simvastatin was formulated as a suspension in 0.5% hydroxypropyl methylcellulose and 0.1% w/v polysorbate 80.

### Animals and treatments

Animal care and experimental procedures were conducted in accordance with the Guide for Animal Experiments by the Korean Academy of Medical Sciences. Female Wistar rats (6 weeks) were obtained from ORIENT-BIO Laboratory Animal Research Center Co., Ltd. (Gyeonggi-do, Korea). Animals were kept on standard rat chow with free access to tap water in a temperature- and humidity-controlled animal house under 12 h light–dark cycles. Eighteen rats were divided into two groups (control and simvastatin groups). The simvastatin group (n = 12) was orally administered simvastatin (formulated as a suspension in 0.5% hydroxypropyl methylcellulose and 0.1% w/v polysorbate 80) at a dose of 80 mg/kg of body weight for 10 days. The control group (n = 6) was treated with volumes of vehicle solution (0.5% hydroxypropyl methylcellulose and 0.1% w/v polysorbate 80) equivalent to those given to the simvastatin group. Individual pre-dose (24 h) and post-dose (3, 6 and 10 days) urine samples were collected into an ice-cooled jar on the metabolic cages. The pooled urine samples were frozen and stored at −80°C for subsequent analysis. All animal experiments were conducted at the Inha University Medical School Animal Experiment Center (Incheon, Korea). At the end of the 10 days, all rats were sacrificed by ether anesthesia. Blood samples for biochemical analyses were obtained from the hearts. The liver specimens were immediately fixed in 10% neutral buffered formalin for histopathological studies.

### NMR Spectroscopic Analysis of Urine

For NMR analysis, urine samples were thawed at room temperature, and 500 µl of urine was mixed with 50 µl of phosphate buffer (1.5 M K_2_HPO_4_/1.5 M NaH_2_PO_4_, pH = 7.4). Samples were allowed to stand for 10 min and then centrifuged at 13,000 rpm for 10 min to remove insoluble material. 500 µl of supernatants were placed in a 5 mm NMR tube containing 50 µl D_2_O and sodium-3-trimethylsily-[2,2,3,3-^2^H_4_]-1-propionate (TSP, 0.025%, w/v) as internal standard reference (0.00 ppm).

### Biochemical analyses of serum

Blood was drawn into lithium heparin tubes after euthanasia for evaluation of serum alanine transaminase (ALT), aspartate transaminase (AST), and plasma creatinine kinase (CK) activity (spectrophotometric assay). Out of the 12 simvastatin-treated animals, we could not obtain the blood for one animal due to its death during the collection procedure. Therefore, biochemical markers were obtained for 11 animals. All biomarkers were analyzed at Inha University Hospital (Incheon, Korea).

### Liver histopathology

After fixation for 48 hours, liver tissues were embedded in paraffin according to routine procedures. Five-µm thick sections were cut and stained with hematoxylin-eosin (H&E) for histopathological evaluation. Two expert pathologists at Inha University Hospital blindly analyzed the tissue slices.

### NMR measurement

One-dimensional spectra of the urine samples were measured with an NMR spectrometer (Bruker Biospin, Avance 500) equipped with a triple-resonance cryogenic probe, operating at a proton NMR frequency of 500.13 MHz. We also used 500 MHz machine (VNMRS500) at Varian Inc. Korea's facility for metabolite identification. The acquisition parameters were essentially the same as those previously reported [27], [37], [38], except the number of scans (64). The spectra were referenced against the TSP signal. The metabolites were identified using Chenomx (Spectral database; Edmonton, Alberta, Canada) by fitting the experimental spectra to those in the database. This study was conducted at the NMR facility at the Korea Basic Science Institute, which is supported by the Bio-MR Research Program of the Korean Ministry of Science and Technology (E29070).

### Multivariate data analysis

All the obtained time domain NMR data were Fourier transformed, phase corrected, and baseline corrected manually. The resulting frequency domain data were binned at a 0.043 ppm interval to reduce the complexity of the NMR data for pattern recognition. The signals were normalized against total integration values and 0.025% TSP, and then converted to an ascii text file. The region corresponding to water (4.6–5.0 ppm) was removed from all spectra. The binning, normalization, and conversion were done using Perl software written in-house. The resultant data sets were then imported into SIMCA-P version 11.0 (Umetrics, Umeå, Sweden) and mean-centered with Pareto scaling for multivariate statistical analysis. Orthogonal projections to latent structure-discriminant analysis (OPLS-DA) was performed with one predictive and four orthogonal components.

## Supporting Information

Table S1 **Change of body weight and biochemical parameters of control and simvastatin treatment groups (averages and student's** ***t*** **-test).** Values are expressed as mean ± SD. ALT, alanine aminotransferase; AST, aspartate aminotransferase; CK, creatinine kinase.
(DOC)

Table S2 **Change of body weight and biochemical parameters of each rat after simvastatin treatment (individual values).**
(DOC)
