# Supplementary material for: An Effective Assessment of Simvastatin-Induced Toxicity with NMR-Based Metabonomics Approach
Source: PLoS One. 2011 Feb 22;6(2):e16641. doi: 10.1371/journal.pone.0016641 (PMC3043067; doi:10.1371/journal.pone.0016641)
Supplement: Table S1 — Change of body weight and biochemical parameters of control and simvastatin treatment groups (averages and student's t -test). Values are expressed as mean ± SD. ALT, alanine aminotransferase; AST, aspartate aminotransferase; CK, creatinine kinase. (DOC) [file pone.0016641.s001.doc]

Figure S1.

| **Parameter** | **Groups** | | |
| --- | --- | --- | --- |
|  | **Control**  **(N = 6)** | **Simvastatin**  **(N = 11)** | **P values** |
| **AST (IU/l)** | **107.50 ± 7.78** | **284.17 ± 87.58** | **P** = **0.019** |
| **ALT (IU/l)** | **29.67 ± 1.91** | **115.73 ± 175.57** | **P** = **0.025** |
| **CK (IU/l)** | **789.67 ± 52.10** | **1298.82 ± 396.52** | **P** = **0.166** |
