# Supplementary material for: An Effective Assessment of Simvastatin-Induced Toxicity with NMR-Based Metabonomics Approach
Source: PLoS One. 2011 Feb 22;6(2):e16641. doi: 10.1371/journal.pone.0016641 (PMC3043067; doi:10.1371/journal.pone.0016641)
Supplement: Table S2 — Change of body weight and biochemical parameters of each rat after simvastatin treatment (individual values). (DOC) [file pone.0016641.s002.doc]

Figure S2.

| **No.** | **Change of Body weight (g)** | **AST (IU/l)** | **ALT (IU/l)** | **CK (IU/l)** |
| --- | --- | --- | --- | --- |
| **A-1** | **3** | **113** | **50** | **1026** |
| **A-2** | **-2.5** | **352** | **204** | **850** |
| **A-3** | **-59.5** | **677** | **89** | **768** |
| **A-4** | **-17.5** | **414** | **53** | **458** |
| **A-5** | **-5** | **71** | **27** | **324** |
| **A-6** | **0** | **78** | **39** | **578** |
| **A-7** | **-53.5** | **387** | **46** | **940** |
| **A-8** | **17** | **72** | **35** | **433** |
| **A-9** | **-50** | **434** | **185** | **1692** |
| **A-10** | **-53** | **639** | **284** | **4147** |
| **A-11** | **-60** | **955** | **261** | **3071** |
